# Elevated serum progesterone/ MII oocyte ratio on the day of human chorionic gonadotropin administration can predict impaired endometrial receptivity

**Published:** 2014-06

**Authors:** Abbas Aflatoonian, Robab Davar, Farzaneh Hojjat

**Affiliations:** *Department of Obstetrics and Gynecology, Research and Clinical Center for Infertility, Shahid Sadoughi University of Medical Sciences, Yazd, Iran.*

**Keywords:** *Rise of progesterone*, *P/E2 ratio*, *IVF outcome*

## Abstract

**Background:** Increased serum progesterone on the day of human chorionic gonadotropin administration may affect in vitro fertilization (IVF) outcome.

**Objective: **The aim of this study was to evaluate whether progesterone elevation on the day of human chorionic gonadotropin administration is associated with poor IVF outcome.

**Materials and Methods:** To determine the relationship between serum progesterone on the day of HCG and the outcome of IVF-embryo transfer treatment, 378 infertile patients undergoing IVF-embryo transfer at Yazd Research and Clinical Center for Infertility from October 2009 to March 2011 were prospectively studied.

**Results:** In this study, absolute p-value and P/E_2_ ratio were not a good predictor outcome of in-vitro fertilization but progesterone per metaphase II were predictive of implantation rate and pregnancy rate with statistically significant results but had no effect on the fertilization rate.

**Conclusion:** We suggest avoided the increased progesterone that the cause of advanced endometrial maturation and impaired endometrial receptivity. If the progesterone is greater than 0.32 per oocyte metaphase II, the embryo transfer can be canceled and freezing all embryos for future transfer must be considered, to increase acceptance of the endometrium and thus increase the success rate.

## Introduction

Moderate increase serum progesterone in the peripheral circulation is visible in most superovulated cycles on the day of human chorionic gonadotropin (HCG) administration. There is debate about the origins and clinical significance of elevated serum progesterone. It has been believed that increased Luteinizing hormone (LH) in late follicular phase cause increased progesterone and it was tried to use agonists and antagonists of gonadotropin-releasing hormone to prevent the rise of LH and premature luteinization. Several publications have reported there is the relationship between progesterone concentration at the end of follicular phase and Foliicle-stimulating hormone (FSH) levels during ovarian stimulation. Probably, the source of progesterone is growing follicle by alone FSH without LH (1). Ubaldi *et al* concluded the greater FSH exposure, and its correlation with the progesterone genesis suggested that one of the possible factors inducing premature luteinization is the increased FSH-induced LH receptivity in granulose cells and no adverse effects of premature luteinization on the in vitro fertilization (IVF) and clinical outcome were observed (2). 

FSH acts on granulosa cells, promoting cell division and steroid biosynthesis from cholesterol terminating at progesterone biosynthesis (3). The rate-limiting step in the intrafollicular steroid biosynthesis is the side-chain cleavage process converting cholesterol (27-carbon molecule) to the 21-carbon products pregnenolone and progesterone; granulose cells are very active manufacturers of progesterone, while theca cells also make significant amounts of progesterone (4). Progesterone is further metabolized to androgens by the theca cells under the trophic influence of LH, and this step can only take place in the theca cell compartment. Androgens are subsequently converted to estrogen through aromatization back in the granulose cells (1). Progesterone produced by the granulosa cells under FSH drive must pass to the vascularized theca cell compartment to be catabolized to androgens. It is probable that the greater the LH drive to the theca cells. The more progesterone catabolism to androgens will take place, leaving fewer products to find its way into the general circulation (1). 

It is postulated that all three factors examined the number of follicles, the FSH drive and the LH activity-influence the concentration of progesterone in the circulation during the follicular phase of ovarian stimulation. The follicle number and FSH concentrations appear to have a positive association with raised progesterone output (5). Because in polycystic ovary syndrome (PCOS) that is a large number of developing follicle's rises progesterone levels and due to use of gonadotropin-releasing hormone antagonist, less premature luteinization in patients with PCOS. Prapas *et al* believed that GnRH antagonist administration during the proliferative phase at a dose of 0.25 mg per day does not appear to adversely affect endometrial receptivity (6-8). Whereas Rackow *et al* believed that use of GnRH antagonists may be associated with impaired HOXA10 expression in endometrial stromal cells and thus may affect endometrial receptivity (9). 

Steward RG and colleagues believed that GnRH antagonists can decrease the rate of premature luteinization, but appear to have no effect on pregnancy (10). The consequences of this premature elevation of serum progesterone on IVF outcome remain controversial (11). Several authors did not find any negative effect of this on IVF outcome (12-20). Other authors reported that pregnancy rate has been inversely related to serum progesterone levels on the day of HCG administration (5, 21-42). 

The objective was to evaluate whether progesterone elevation on the day of human chorionic gonadotropin administration is associated with the IVF outcome.

## Materials and methods

**Patients**

In this retrospective study, we analyzed the results of 378 women with normal ovaries participating in an IVF program-embryo transfer in Research and Clinical Center for Infertility, Yazd from October 2009 to March 2011. Who retrieved their eggs and performed embryo transfer and younger than 40 years with FSH on the 3^rd^ day cycles less than 10 IU/L and E_2_ on the 3^rd^ day cycles less than 80 P/ml included in this study. Women with more than 40 years of age or with FSH on the 3^rd^ day cycles more than 10 IU/L or egg donors or no documented FSH and E_2_ in the 3^rd^ day of the cycle excluded in this study. The article has been approved by Research and Clinical Center for Infertility Ethical Committee.

**Treatment protocol**

Three standard protocols for ovarian stimulation were used for all patients: 1) GnRH agonist protocol (long luteal protocol) 2) GnRH antagonist protocol (flexible protocol) 3) microdose protocol. From 378 patients, were treated 168 patients with GnRH agonist protocol, 160 patients with GnRH antagonist protocol and 50 patients with microdose protocol (Table І). Final maturation of the oocytes was effected with 10000 IU HCG when there were at least two follicles 16 mm. Ovum retrieval was performed 32-36h after HCG administration by vaginal ultrasound, and embryo transferred 48 h later. 

**Assays**

Age, treatment protocol, basal hormone levels: FSH, LH, FSH/LH, estradiol (E_2_), progesterone (P), and type of gonadotropin was recorded (Table II). In the day of HCG administration, the endometrial thickness (mm) measured and the endometrial patterns evaluated. E_2_ (P/mL) and P (ng/mL) measured on the day of hCG administration and P/E_2_ ratio (P[ng/mL]×1000/E_2_[P/mL]) and P/mature oocyte calculated. 

Numbers of mature oocyte after retrieval of the ovum counted. All the cycles were grouped according to the serum progesterone concentration and P/E_2_ ratio and P/mature oocyte ratio on the day of hCG administration. According to previous studies premature luteinization defined as serum P and P/E_2_ ratio ≥1 (46, 71, 72) and cut off for the P/mature oocyte ratio calculated (p/mature oocyte >0.32). Then impact of these hormonal ratios on fertilization rate, implantation rate and pregnancy rate were evaluated.

**Statistical analysis**

After data collection and coding, enter them into the computer and using SPSS software (Statistical Package for the Social Sciences, version 18.0, SPSS Inc, Chicago, Illinois, USA) and Mann-whitney, Chi-square, Independent-samples T-test, analysis the results. We determine the normal distribution of data by Kolmogorov-Simron z test. Our statistical significant was set at p<0.05.

## Results

In this study, all the cycles were in two groups according to serum progesterone concentration, P/E_2_ ratio and P/mature oocyte. There were no significant differences in age (year), basal FSH (IU/L), basal LH (IU/L), FSH/LH, basal E_2_ (P/ml), basal progesterone (ng/ml) in two groups (Table II). In the group with the serum progesterone level <1, fertilization rate=59.5±8%, implantation rate=24.4±25%, chemical pregnancy=52±5% and in the group with the serum progesterone level ≥1, fertilization rate=58±10%, implantation rate=22.7±32%, chemical pregnancy=44±5% as a result fertilization rate, implantation rate and pregnancy rate no significant difference between two groups. 

And in the patients with P/E_2_ ratio <1 were fertilization rate=58.7±8%, implantation rate=23.8%±27, chemical pregnancy=48±5% and in the patients with P/E_2_ ratio≥1: fertilization rate=58.7±11%, implantation rate=22.9±32%, chemical pregnancy=47±5%, fertilization rate, implantation rate and pregnancy rate no significant difference between two groups. In the group with P/mature oocyte ratio≤0.32: fertilization rate=58.8±8.3%, implantation rate=28.5±30%, chemical pregnancy=57±5% and in the group 2 with the P/mature oocyte ratio>0.32: fertilization rate=58.6±12.5%, implantation rate=8±20%, chemical pregnancy=20±4%, fertilization rate no significant difference between two groups despite implantation rate and pregnancy rate significant difference between two groups (Table III). 

Positive relationship between the number of mature oocyte and serum progesterone concentrations was seen. From 378 patients, in 179 cases oocyte's numbers were equal 9.25±2 and progesterone <1 (ng/ml) and in 199 cases numbers of oocyte were equal 11.5±3 and progesterone ≥1 (ng/ml) (p<0.0001). Add FSH without LH increase the serum progesterone concentrations. In 122 patients, alone FSH was used for ovarian stimulation, in 66 cases was the P/MII oocyte ratio >0.32 and in 256 patients, FSH+LH used for ovarian stimulation only in 26 cases P/MII oocyte ratio>0.32 (p<0.0001). Concentrations of progesterone have no effect on endometrial thickness but are effective on endometrial pattern (Table II).

**Table I:** Treatment protocols

**Protocol**	**P/MII oocyte ratio**		**Total**
	**≤0.32**	**>0.32**	
GnRH agonist	130	38	168
GnRH antagonist	136	24	160
Microdose	20	30	50
Total	286	92	378

**Table II:** Demographic characteristics of patients and Effect of addition FSH without LH on serum p-level and progesterone concentration on endometrial thickness and endometrial pattern

**P/MII oocyte ratio**			**Mean**	**p-value**
Age (year)				
		<0.32	31±2.7	0.09
		≥0.32	30±4	
Basal FSH(IU/L)				
		<0.32	7±0.8	0.43
		≥0.32	7±1	
Basal LH(IU/L)				
		<0.32	6.4±0.6	0.11
		≥0.32	6.25±1	
FSH/LH				
		<0.32	1.11	0.27
		≥0.32	1.12	
Basal E_2_ (P/ml)				
		<0.32	58±6	0.11
		≥0.32	56±8.5	
Basal progesterone (ng/ml)				
		<0.32	0.75	0.11
		≥0.32	0.74	
Mean LH day of HCG (IU/L)				
		<0.32	1.4	0.11
		≥0.32	1.36	
Ovarian stimulation with FSH alone				
	<0.32		56	<0.0001
	≥0.32		66	
Ovarian stimulation with FSH+LH				
	<0.32		230	-
	≥0.32		26	
Endometrial thickness (mm)				
	≤ 0.32		9.17±0.5	0.39
	> 0.32		9.21±0.4	
Triple line (%)				
	≤ 0.32		66±5	<0.0001
	> 0.32		13±3	
Echogen (%)				
	≤ 0.32		34±5	<0.0001
	> 0.32		87±3	

**Table III:** Effect of serum p level, P/E_2_ ratio and P/mature on fertilization, implantation, and pregnancy rates

**variable**	**P**		**p-value**	**P/E**_2_ ** ratio**		**p-value**	**P/MII oocyte ratio**		**p-value**
	**<1 (n=179)**	**≥1 (n=199)**		**<1 (n=248)**	**≥1 (n=130)**		**≤ 0.32 (n=286)**	**> 0.32 (n=92)**	
Fertilization rate (%)	59.5±8	58±10	0.18	58.7±8	58.7±11	0.60	58.8±8	58.6±12	0.51
Implantation rate (%)	24.4±25	22.7±32	0.58	23.8±27	22.9±32	0.76	28.5±30	8±20	<0.0001
Chemical pregnancy (%)	52±5	44±5	0.13	48±5	47±5	0.84	57±5	20±4	<0.0001

## Discussion

In this study, we evaluate the effect of elevated serum progesterone in the day HCG administration in the outcome of IVF cycles. Success of IVF cycle was dependent to number and quality of oocytes and endometrial receptivity. Several authors reported that outcome of IVF has been inversely related to serum progesterone levels on the day of HCG administration (5, 21-43). In our study absolute p-value and P/E_2_ ratio were not a good predictor outcome of in-vitro fertilization. "Tavaniotou *et al* in 2003 were concluded endometrial integrin expression is more consistently present in the early luteal phase in stimulated cycles than in natural cycles, and this may be related to the higher serum progesterone concentration and/or the more advanced endometrial histological features" (27). 

Bourgain *et al* in 2003 were concluded the endometrium in IVF cycles have shown premature secretory changes in the post-ovulatory and early luteal phase of IVF cycles, followed by a large proportion of dyssynchronous glandular and stromal differentiation in the mid-luteal phase. "These findings suggest a profound modification of luteal endometrial development in stimulated cycles. This hypothesis is further supported by the demonstration of a modified endometrial steroid receptor regulation and a profound antiproliferative effect in IVF cycles. The time of maximal endometrial receptivity is defined as the implantation window and is characterized by the expression of various endometrial products, among which pinopodes, integrins and leukemia inhibitory factors are best described. 

Premature expression of pinopodes and integrins are in line with the observation of precocious luteal transformation following ovarian stimulation. Studies exploring the endometrium within the cycle of embryo transfer have shown a deleterious effect of severe peri-ovulatory maturation advancement exceeding three days, as no clinical pregnancies were obtained in this condition (28). Azem *et al* in 2008, our data demonstrate high serum P adversely affects implantation and pregnancy rates" (30).

Kilicdag *et al* in 2010 were concluded elevated serum progesterone levels on the day of HCG administration were associated with diminished implantation rates and live birth rates regardless of ovarian reserve (34). Bosch *et al* in 2011 were concluded that the elevated P levels may have a dual influence on pregnancy rate. One related to endometrial receptivity (37). Li *et al* in 2011 from micro RNA and microarray analysis suggests dissimilar endometrial receptivity in patients with high P levels on the day of HCG, and had poor pregnancy rates (41). 

In this study since high levels of progesterone no effect on the fertilization rate while significantly reduced the implantation rate, can be concluded that high levels of progesterone, reduce the endometrial receptivity. According to previous studies because the main sources of productive progesterone are follicles, in effect of FSH, the level of progesterone produced per mature oocyte is the better predictor for IVF results (1). In our study the positive relationship seen between the number of mature oocyte and serum progesterone concentrations. In this study from 50 patients were treated with microdose protocol 30 patients had a P/MII oocyte ratio>0.32, despite from 160 patients were treated with GnRH antagonist protocol 24 patients had a P/MII oocyte ratio>0.32 shows if adding the GnRH antagonist can be decreased the LH surge and progesterone levels. 

"The abrupt pituitary suppression that rapidly follows administration of the GnRH antagonist, occurs too late in the cycle of stimulation to suppress LH before it begins adversely affect follicle, egg and endometrium development. Low-dose HCG alone in the late COH stages avoid increasing progesterone. Patients received ovarian priming with recombinant FSH/HMG followed by low-dose HCG (200 IU/day) alone" (46). The addition of LH (HCG) to the stimulation protocol results in a higher yield of mature oocytes, excellent-quality embryos and increase endometrial receptivity (47). Interests this protocol was included: I) more catabolism the progesterone to androgen in follicular fluid due to increased LH a result less appears progesterone in the blood circulation, II) reduced recombinant FSH/HMG consumption III) reduced number of small preovulatory follicles, IV) more estrogenic intrafollicular environment thereupon to estrogen formed from androgen created as a result of more catabolism the progesterone due to LH. V) Higher fertilization rate 

The use of a less aggressive agonist treatment such as intranasal Nafarelin, which has been shown to reduce the demand for FSH injections, may also reduce the incidence/degree of profound LH suppression, and the consequences reduced the levels of circulating progesterone (48). Flexible antagonist protocol with Low-dose HCG alone started simultaneously starting antagonist: in IVF-ICSI patients undergoing COS with the antagonist protocol, significantly increases pregnancy rates and reduces the incidence of premature luteinization (49). 

The antagonist administration was initiated according to at least one of the following patient-specific criteria: (i) At least, one follicle measuring >14 mm; (ii) estradiol levels >600 P/ml; (iii) LH levels >10 IU/l (50). "Use of Mifepristone, COH is associated with advanced endometrial histology and relatively high p-levels in the late follicular phase occurring in a relatively large proportion of IVF cycles despite GnRH analog treatment, which is associated with impaired implantation and lower pregnancy rates. Low dosages of mifepristone have been shown to delay endometrial maturation" (51).

Mifepristone and FSH were administered daily from the beginning of the COH and concomitantly with an intra-muscular injection of 50 mg progesterone to reverse residual antiprogestogenic activity of mifepristone on the day of HCG administration (52). If the serum progesterone level more than 0.32 per metaphase II oocytes, the embryo transfer canceled and was considered freezing for all embryos to future transfer.

## Conclusion

We suggest to avoid the increased progesterone that the cause of advanced endometrial maturation and impaired endometrial receptivity administration Low-dose HCG alone in the late COH stages and the use of a less aggressive agonist treatment and in flexible antagonist protocol administration, Low-dose HCG alone simultaneously starting antagonist and adding the GnRH antagonist with microdose protocol and use of Mifepristone daily from the beginning of the COH. If the progesterone greater than 0.32 per oocyte metaphase the better embryo transfer canceled and considered freezing all embryos for future transfer. To increase acceptance of the endometrium and thus increase the success rate.

## Footenote

***This article extracted from fellowship course thesis. (Farzaneh Hojjat)***

## References

[1] Fleming R, Jenkins J (2010). The source and implications of progesterone rise during the follicular phase of assisted reproduction cycles. Reprod Biomed Online.

[2] Ubaldi F, Camus M, Smitz J, Bennink HC, Steirteghem AV, Devroey P (1996). Premature luteinization in in-vitro fertilization cycles using gonadotropin-releasing hormone agonist (GnRH-a) and recombinant follicle-stimulating hormone (FSH) and GnRH-a and urinary FSH. Fertil Steril.

[3] Fleming R (2008). Progesterone elevation on the day of hCG: methodological issues. Hum Reprod Update.

[4] Moon YS, Tsang BK, Simpson C, Armstrong DT (1978). 17 beta-Estradiol biosynthesis in cultured granulosa and thecal cells of human ovarian follicles: stimulation by follicle-stimulating hormone. J Clin Endocrinol Metab.

[5] Andersen AN, Devroey P, Arce JC (2006). Clinical outcome following stimulation with highly purified hMG or recombinant FSH in patients undergoing IVF: a randomized assessor-blind controlled trial. Hum Reprod.

[6] Ertunc D, Tok EC, Savas A, Ozturk I, Dilek S (2010). Gonadotropin-releasing hormone antagonist use in controlled ovarian stimulation and intrauterine insemination cycles in women with polycystic ovary syndrome. Fertil Steril.

[7] Stadtmauer LA, Sarhan A, Duran EH, Beydoun H, Bocca S, Pultz B (2011). The impact of a gonadotropin-releasing hormone antagonist on gonadotropin ovulation induction cycles in women with polycystic ovary syndrome: a prospective randomized study. Fertil Steril.

[8] Prapas N, Tavaniotou A, Panagiotidis Y, Prapa S, Kasapi E, Goudakou M (2009). GnRH antagonists and endometrial receptivity in oocyte recipients: a prospective randomized trial. Reprod Biomed Online.

[9] Rackow BW, Kliman HJ, Taylor HS (2008). GnRH antagonists may affect endometrial receptivity. Fertil Steril.

[10] Steward RG, Gill I, Williams DB, Witz CA, Griffith J, Haddad GF (2011). Cetrorelix lowers premature luteinization rate in gonadotropin ovulation induction-intrauterine insemination cycles: a randomized-controlled clinical trial. Fertil Steril.

[11] Elnashar A (2010). Progesterone rise on the day of HCG administration (premature luteinization) in IVF: An overdue update. J Assist Reprod Genet.

[12] Antoine JM, Firmin C, Salat-Baroux J, Alvarez S, Cornet D, Mandelbaum J (1992). [Prognostic value of preovulatory elevations of plasma progesterone during in vitro fertilization using LHRH agonists in a long protocol]. J Gynecol Obstet Biol Reprod (Paris).

[13] Levy MJ, Smotrich DB, Widra EA, Sagoskin AW, Murray DL, Hall JL (1995). The predictive value of serum progesterone and 17-OH progesterone levels on in-vitro fertilization outcome. J Assist Reprod Genet.

[14] Hofmann GE, Khoury J, Michener C (2002). Elevated serum progesterone-to-estradiol ratio during gonadotropin stimulation for intrauterine insemination or in-vitro fertilization is not associated with diminished ovarian reserve. Fertil Steril.

[15] Seow KM, Lin YH, Huang LW, Hsieh BC, Huang SC, Chen CY (2007). Subtle progesterone rise in the single-dose gonadotropin-releasing hormone antagonist (cetrorelix) stimulation protocol in patients undergoing in-vitro fertilization or intracytoplasmic sperm injection cycles. Gynecol Endocrinol.

[16] Venetis CA, Kolibianakis EM, Papanikolaou E, Bontis J, Devroey P, Tarlatzis BC (2007). Is progesterone elevation on the day of human chorionic gonadotrophin administration associated with the probability of pregnancy in in-vitro fertilization? A systematic review and meta-analysis. Hum Reprod Update.

[17] Nikolettos N, Asimakopoulos B, Koster F, Schopper B, Schulz C, Caglar GS (2008). Cytokine profile in cases with premature elevation of progesterone serum concentrations during ovarian stimulation. Physiol Res.

[18] Saleh HA, Omran MS, Draz M (2009). Does subtle progesterone rise on the day of HCG affect pregnancy rate in long agonist ICSI cycles?. J Assist Reprod Genet.

[19] Segal S, Glatstein I, McShane P, Hotamisligil S, Ezcurra D, Carson R (2009). Premature luteinization and in-vitro fertilization outcome in gonadotropin/ gonadotropin-releasing hormone antagonist cycles in women with polycystic ovary syndrome. Fertil Steril.

[20] Sönmezer M, Pelin Cil A, Atabekoglu C, Özkavukçu S, Özmen B (2009). Does premature luteinization or early surge of LH impair cycle outcome? Report of two successful outcomes. J Assist Reprod Genet.

[21] Garcia JE, Acosta AA, Hsiu JG, Jones HW Jr (1984). Advanced endometrial maturation after ovulation induction with human menopausal gonadotropin/ human chorionic gonadotropin for in vitro fertilization. Fertil Steril.

[22] Silverberg KM, Burns WN, Olive DL, Riehl RM, Schenken RS (1991). Serum progesterone levels predict success of in-vitro fertilization/embryo transfer in patients stimulated with leuprolide acetate and human menopausal gonadotropins. J Clin Endocrinol Metab.

[23] Fanchin R, de Ziegler D, Taieb J, Hazout A, Frydman R (1993). Premature elevation of plasma progesterone alters pregnancy rates of in-vitro fertilization and embryo transfer. Fertil Steril.

[24] Burns WN, Witz CA, Klein NA, Silverberg KM, Schenken RS (1994). Serum progesterone concentrations on the day after human chorionic gonadotropin administration and progesterone/oocyte ratios predict in-vitro fertilization/embryo transfer outcome. J Assist Reprod Genet.

[25] Sims JA, Seltman HJ, Muasher SJ (1994). Early follicular rise of serum progesterone concentration in response to a flare-up effect of gonadotrophin-releasing hormone agonist impairs follicular recruitment for in-vitro fertilization. Hum Reprod.

[26] Harada T, Yoshida S, Katagiri C, Takao N, Ikenari T, Toda T (1995). Reduced implantation rate associated with a subtle rise in serum progesterone concentration during the follicular phase of cycles stimulated with a combination of a gonadotrophin-releasing hormone agonist and gonadotrophin. Hum Reprod.

[27] Tavaniotou A, Bourgain C, Albano C, Platteau P, Smitz J, Devroey P (2003). Endometrial integrin expression in the early luteal phase in natural and stimulated cycles for in-vitro fertilization. Eur J Obstet Gynecol Reprod Biol.

[28] Bourgain C, Devroey P (2003). The endometrium in stimulated cycles for IVF. Hum Reprod Update.

[29] Ozcakir HT, Levi R, Tavmergen E, Goker EN (2004). Premature luteinization defined as progesterone estradiol ratio >1 on hCG administration day seems to adversely affect clinical outcome in long gonadotropin-releasing hormone agonist cycles. J Obstet Gynaecol Res.

[30] Azem F, Tal G, Lessing JB, Malcov M, Ben-Yosef D, Almog B (2008). Does high serum progesterone level on the day of human chorionic gonadotropin administration affect pregnancy rate after intracytoplasmic sperm injection and embryo transfer?. Gynecol Endocrinol.

[31] Lai TH, Lee FK, Lin TK, Horng SG, Chen SC, Chen YH (2009). An increased serum progesterone-to-estradiol ratio on the day of human chorionic gonadotropin administration does not have a negative impact on clinical pregnancy rate in women with normal ovarian reserve treated with a long gonadotropin releasing hormone agonist protocol. Fertil Steril.

[32] Lee FK, Lai TH, Lin TK, Horng SG, Chen SC (2009). Relationship of progesterone/estradiol ratio on day of hCG administration and pregnancy outcomes in high responders undergoing in-vitro fertilization. Fertil Steril.

[33] Polotsky AJ, Daif JL, Jindal S, Lieman HJ, Santoro N, Pal L (2009). Serum progesterone on the day of human chorionic gonadotropin administration predicts clinical pregnancy of sibling frozen embryos. Fertil Steril.

[34] Kilicdag EB, Haydardedeoglu B, Cok T, Hacivelioglu SO, Bagis T (2010). Premature progesterone elevation impairs implantation and live birth rates in GnRH-agonist IVF/ICSI cycles. Arch Gynecol Obstet.

[35] Lai TH, Lin TK, Lee FK (2010). Reply of the Authors: Premature luteinization defined by an increased progesterone/ estradiol ratio on day of human chorionic gonadotropin administration is a manifestation of diminished ovarian responsiveness to controlled ovarian hyperstimulation. Fertil Steril.

[36] Younis JS, Ben-Shlomo I, Ben-Ami M (2010). Premature luteinization defined by an increased progesterone/ estradiol ratio on day of human chorionic gonadotropin administration is a manifestation of diminished ovarian responsiveness to controlled ovarian hyperstimulation. Fertil Steril.

[37] Bosch E (2011). Reply: Elevated P level on the day of hCG administration is related to FSH dose: is it the whole truth?. Hum Reprod.

[38] Cakmak H, Taylor HS (2011). Implantation failure: molecular mechanisms and clinical treatment. Hum Reprod Update.

[39] Elgindy EA (2011). Progesterone level and progesterone/estradiol ratio on the day of hCG administration: detrimental cutoff levels and new treatment strategy. Fertil Steril.

[40] Labarta E, Martínez-Conejero JA, Alamá P, Horcajadas JA, Pellicer A, Simón C (2011). Endometrial receptivity is affected in women with high circulating progesterone levels at the end of the follicular phase: a functional genomics analysis. Hum Reprod.

[41] Li R, Qiao J, Wang L, Li L, Zhen X, Liu P (2011). MicroRNA array and microarray evaluation of endometrial receptivity in patients with high serum progesterone levels on the day of hCG administration. Reprod Biol Endocrinol.

[42] Van Vaerenbergh I, Fatemi HM, Blockeel C, Van Lommel L, In't Veld P, Schuit F (2011). Progesterone rise on HCG day in GnRH antagonist/rFSH stimulated cycles affects endometrial gene expression. Reprod Biomed Online.

[43] Papanikolaou EG, Kolibianakis EM, Pozzobon C, Tank P, Tournaye H, Bourgain C (2009). Progesterone rise on the day of human chorionic gonadotropin administration impairs pregnancy outcome in day 3 single-embryo transfer, while has no effect on day 5 single blastocyst transfer. Fertil Steril.

[44] Younis JS, Matilsky M, Radin O, Ben-Ami M (2001). Increased progesterone/estradiol ratio in the late follicular phase could be related to low ovarian reserve in in-vitro fertilization-embryo transfer cycles with a long gonadotropin-releasing hormone agonist. Fertil Steril.

[45] Ou YC, Lan KC, Chang SY, Kung FT, Huang FJ (2008). Increased Progesterone/Estradiol Ratio on the Day of hCG Administration Adversely Affects Success of In-Vitro Fertilization-Embryo Transfer in Patients Stimulated with Gonadotropin-releasing Hormone Agonist and Recombinant Follicle-stimulating Hormone. Taiwan J Obstet Gynecol.

[46] Filicori M, Cognigni GE, Gamberini E, Parmegiani L, Troilo E, Roset B (2005). Efficacy of low-dose human chorionic gonadotropin alone to complete controlled ovarian stimulation. Fertil Steril.

[47] Huddleston HG, Jackson KV, Doyle JO, Racowsky C (2009). hMG increases the yield of mature oocytes and excellent-quality embryos in patients with a previous cycle having a high incidence of oocyte immaturity. Fertil Steril.

[48] Lockwood GM, Pinkerton SM, Barlow DH (1995). Endocrinology: A prospective randomized single-blind comparative trial of nafarelin acetate with buserelin in long-protocol gonadotrophin-releasing hormone analogue controlled in-vitro fertilization cycles. Hum Reprod.

[49] Bakas P, Konidaris S, Liapis A, Gregoriou O, Tzanakaki D, Creatsas G (2011). Role of gonadotropin-releasing hormone antagonist in the management of subfertile couples with intrauterine insemination and controlled ovarian stimulation. Fertil Steril.

[50] Lainas T, Zorzovilis J, Petsas G, Stavropoulou G, Cazlaris H, Daskalaki V (2005). In a flexible antagonist protocol, earlier, criteria-based initiation of GnRH antagonist is associated with increased pregnancy rates in IVF. Hum Reprod.

[51] Batista MC, Cartledge TP, Zellmer AW, Merino MJ, Axiotis C, Loriaux DL (1992). Delayed endometria maturation induced by daily administration of the antiprogestin RU 486: a potential new contraceptive strategy. Am J Obstet Gynecol.

[52] Escudero EL, Boerrigter PJ, Bennink HJ, Epifanio R, Horcajadas JA, Olivennes F (2005). Mifepristone is an effective oral alternative for the prevention of premature luteinizing hormone surges and/or premature luteinization in women undergoing controlled ovarian hyperstimulation for in-vitro fertilization. J Clin Endocrinol Metab.

